# Neuroergonomic assessment of developmental coordination disorder

**DOI:** 10.1038/s41598-022-13966-9

**Published:** 2022-06-17

**Authors:** Shawn Joshi, Benjamin D. Weedon, Patrick Esser, Yan-Ci Liu, Daniella N. Springett, Andy Meaney, Mario Inacio, Anne Delextrat, Steve Kemp, Tomás Ward, Hooshang Izadi, Helen Dawes, Hasan Ayaz

**Affiliations:** 1 School of Biomedical Engineering, Science and Health Systems, Drexel University, Philadelphia, PA USA; 2 College of Medicine, Drexel University, Philadelphia, PA USA; 3 Centre for Movement, Occupation and Rehabilitation Services, Oxford Brookes University, Oxford, UK; 4 Nuffield Department of Clinical Neurology, University of Oxford, Oxford, UK; 5 School and Graduate Institute of Physical Therapy, College of Medicine, National Taiwan University, Taipei, Taiwan; 6 Department for Health, University of Bath, Bath, UK; 7 Research Center in Sports Sciences, Health Sciences and Human Development, University of Maia, Porto, Portugal; 8 Insight SFI Research Centre for Data Analytics, Dublin City University, Dublin, Ireland; 9 School of Engineering, Computing and Mathematics, School of Technology, Design and Environment, Oxford Brookes University, Oxford, UK; 10 Department of Psychological and Brain Sciences, College of Arts and Sciences, Drexel University, Philadelphia, PA USA; 11 Drexel Solution Institute, Drexel University, Philadelphia, PA USA; 12 Department of Family and Community Health, University of Pennsylvania, Philadelphia, PA USA; 13 Center for Injury Research and Prevention, Children’s Hospital of Philadelphia, Philadelphia, PA USA; 14 Intersect@Exeter, College of Medicine and Health, University of Exeter, Exeter, UK; 15 Oxford Health BRC, University of Oxford, Oxford, UK; 16 NHS Foundation Trust, Oxford University Hospitals, Oxford, UK; 17 Physical Therapy Center, National Taiwan University Hospita, Taipei, Taiwan

**Keywords:** Predictive markers, Brain imaging, Neurodevelopmental disorders, Biomedical engineering, Rehabilitation, Paediatric research, Cognitive neuroscience

## Abstract

Until recently, neural assessments of gross motor coordination could not reliably handle active tasks, particularly in realistic environments, and offered a narrow understanding of motor-cognition. By applying a comprehensive neuroergonomic approach using optical mobile neuroimaging, we probed the neural correlates of motor functioning in young people with Developmental Coordination Disorder (DCD), a motor-learning deficit affecting 5–6% of children with lifelong complications. Neural recordings using fNIRS were collected during active ambulatory behavioral task execution from 37 Typically Developed and 48 DCD Children who performed cognitive and physical tasks in both single and dual conditions. This is the first of its kind study targeting regions of prefrontal cortical dysfunction for identification of neuropathophysiology for DCD during realistic motor tasks and is one of the largest neuroimaging study (across all modalities) involving DCD. We demonstrated that DCD is a motor-cognitive disability, as gross motor /complex tasks revealed neuro-hemodynamic deficits and dysfunction within the right middle and superior frontal gyri of the prefrontal cortex through functional near infrared spectroscopy. Furthermore, by incorporating behavioral performance, decreased neural efficiency in these regions were revealed in children with DCD, specifically during motor tasks. Lastly, we provide a framework, evaluating disorder impact in ecologically valid contexts to identify when and for whom interventional approaches are most needed and open the door for precision therapies.

## Introduction

Functional deficits in motor skill acquisition and execution are common concerns regarding child development, and serious impairments can be characterized as Developmental Coordination Disorder (DCD)^1,2^. DCD is a motor-cognitive deficit prevalent among 5–6% of school-aged children (a disorder of high prevalence) often manifested as clumsiness, slowness, and poor motor skill acquisition leading to lifelong impacts within personal, social, academic, and occupational functioning^2,3^. DCD is not a condition that children simply “outgrow,” as one in two children diagnosed with DCD retain persistent negative impacts, even upwards of 10 years later^4^. There is currently no cure for DCD, and early pharmacological and non-pharmacological interventions have the potential to reduce the emotional, physical, social and economic consequences that are often associated with this disorder^2,4–6^.

It has been purported that DCD as a neurodevelopmental disorder, may have identifiable neuropathology, and while traditional functional neuroimaging has been critical in understanding the neural mechanisms of a variety of complex disorders^7^ including DCD^1,8,9^, their use in motor-cognitive research and understanding has been limited to stationary fine motor tasks and is not reflective of motor disability within active, ecologically relevant contexts^1,8–11^. This is particularly evident in DCD research, which when more traditionally studied under rigid, confined, and unrealistic conditions, have resulted in variable conclusions of functional neuropathology^1,8,9,11^, and different cortical activation patterns between DCD children and their Typically Developed (TD) counterparts^1,9^. Seeing as DCD is a motor-related disorder, contextually relevant neuroimaging techniques such as functional near infrared spectroscopy (fNIRS) have become increasingly more important tools in its study and the study of sensorimotor control mechanisms ^12–14^.

Sensorimotor control includes motor cognition, the cognitive processing that controls and modulates complex motor outputs, including planning, preparation, and motor production^15,16^. It is responsible for movement control and coordination, localized and specialized within numerous interconnected brain regions^17,18^. A critical importance is placed on the prefrontal cortex (PFC) in the study of motor cognition, as it is an integral regulator of these complex pathways involving motor behavior, executive functioning, sensory processing, sustained attention, and future planning^19–22^. The motor network is densely interconnected within the PFC, particularly within the dorsolateral (dl) and ventrolateral (vl) PFC^23^. The dlPFC is known for the cognitive control in task planning, sensorimotor plasticity, and learning of motor action sequences^18,24–27^. The vlPFC is involved with the initiation and control of voluntary movements^17,18^ and is associated with visuomotor processing, action inhibition, and external integration^18,24,25^.

As the PFC is involved in both higher-order motor and cognitive domains, impairments in cognitive processing of the PFC have been associated with impaired motor execution^28–31^. Additionally, many developmental disabilities often disrupt these executive functions, particularly within the frontal lobes, impeding processing of relevant sensory information, resulting in clumsiness or incoordination^15,32^. The complex nature of how these higher-level brain areas are activated during complex motor execution is unclear, and therefore the study of the PFC within motor-cognition and DCD is important within complex motor execution and motor learning.

The study of both acquired and inherited structural and functional impairments of the PFC can be helpful in understanding DCD. Both children and adults with acquired conditions including concussion^33–35^ and mild TBI^36^ reported hypoperfusion, or lower cerebral hemodynamics through fMRI and fNIRS compared to healthy controls during cognitive tasks (i.e. neurocognitive test batteries [memory recall, color Stroop, etc.] evaluating verbal/visual memory, processing speed, reaction time, distraction etc.). During physical tasks, inherited conditions including Down’s Syndrome^37^, also revealed significantly reduced PFC activity within fine motor tasks. These findings discuss the conditions neurally, evaluating neural resources (i.e. Oxygenated Hemoglobin [HbO]) without behavioral context.

However, to maintain motor and cognitive performance, acquired conditions such as stroke^38^, and multiple sclerosis^39^ and other natural/non-pathological conditions including increased age^29,32^, detail the individual’s need for enhanced brain activity and or recruitment from other areas of the PFC as it related to dual-task impairment. Similarly, as found in typical gait, aging, pathology and dual task conditions lead to altered PFC activity, as the attentional load requires recruitment of multiple cortical areas and are spread from the motor cortex^40,41^. These findings represent interpretations of neurobehavioral results and are examples of conditions that lead to neurally inefficiency, as greater neural resources are required while still leading to equal or poorer performance to typical healthy controls^42–44^.

Heterogenous PFC dysfunction has been found in most MRI studies of DCD (often with co-morbid conditions including Attention-Deficit Hyperactivity Disorder, Autism Spectrum Disorder, etc.) detailing atypical structural, processing, and hypofunctioning^45^ during stationary tasks^8^. Additionally, EEG has indicated under-activation of the PFC for DCD children, with enhanced activation in structures outside the Mirror Neuron System^1^. This may be due to the inability or deficiency in automatization of physical task learning within DCD^46^, as is further detailed in the impaired performance and executive processing during dual tasking (simultaneous motor and cognitive tasks)^30,47,48^. Drawing observations from these studies regarding an array of disability conditions and specifically that of DCD, we hypothesized that 1) behaviorally, DCD children would have impaired performance compared to their TD counterparts of their Physical Performance (PhysP), as well as their Cognitive Performance (CogP) particularly during dual-tasking (with the addition of a motor-element), 2) neurally, DCD children would indicate hypo-functioning of the PFC during motor and dual tasks, however 3) neurobehaviorally, DCD children would be expected to complete tasks in a neurally inefficient manner, and thus need to utilize more cognitive resources to maintain CogP and PhysP. We expect that in tasks without any motor element, TD and DCD children will behaviorally, and neurally perform similarly.

We thus set out to study motor skill acquisition and performance in a novel dynamic task in young people using fNIRS as the neuroimaging tool alongside motor performance in order to identify motor-cognitive deficits to further understand DCD. This study aimed to demonstrate the feasibility and importance in determining motor-cognitive disorder impact and extent using an sufficiently replicable, and broadly applicable ecologically-valid neuroergonomic approach, using mobile neuroimaging accounting for motion artifacts with advanced statistical processing techniques, incorporating behavioral and brain-based assessments during active motor tasks^10,49^.

## Results

We challenged both DCD children and those with neurotypical development (TD) in separate cognitive and physical tasks, and a combinatory dual task (as shown in movie S1). Our study is the first of its kind in targeting regions of prefrontal cortical dysfunction for identification of neuropathophysiology for DCD during realistic, active ambulatory motor tasks, and is one of the largest neuroimaging study (across all modalities) involving DCD^1,8,11,50^. Therefore, our work is the first to reveal the neural underpinnings of how DCD affects physical activity, and gross motor performance.

### Behavioral results

#### Cognitive performance (CogP)

**Figure 1:**
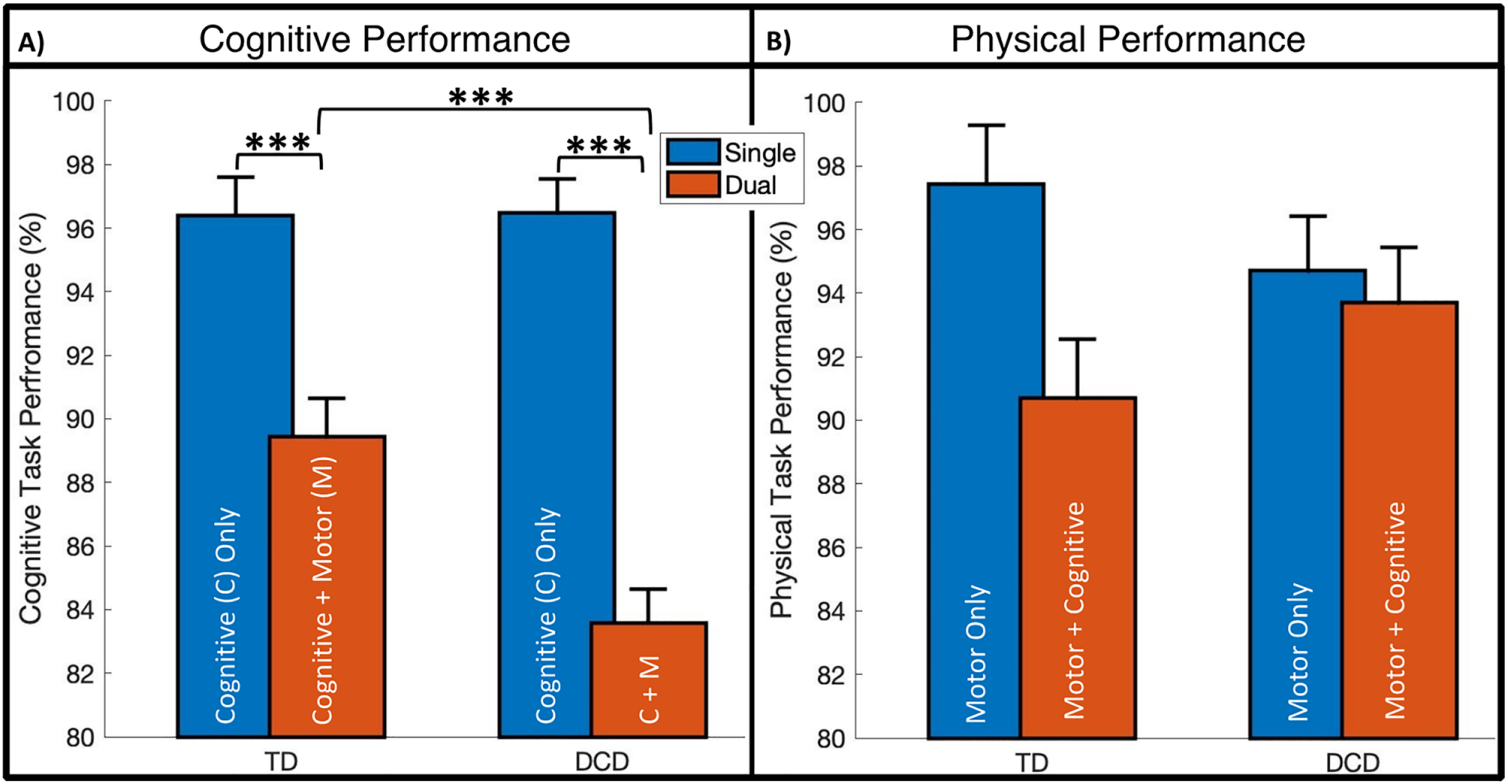
Cognitive Task Performance (%) and Physical Task Performance (%) between group (DCD and TD) per task condition (Single and Dual) for 85 subjects with error bars representing standard error of the mean. Increased values indicate better performance. (*p < 0.05; **p < 0.01; ***p < 0.001). (**A**) Single task is the cognitive only task, while dual task is with the additional motor task. (**B**) Single task is the motor only task, while the dual task is with the additional cognitive task.

Results regarding Cognitive Performance (CogP) indicated significant main effects for Group (F_1,505_ = 6.42, *p* = 0.012*, *d* = *0.34*), between TD and DCD, and Task (F_1,505_ = 76.13, *p* < 0.001***, *d* = *0.82*), between Single and Dual. A statistically significant interaction effect between Group and Task was found within CogP (F_1,505_ = 6.81, *p* = 0.009**), as depicted in Fig. 1A.

In evaluating the interaction effects of Group and Task for CogP (see Fig. 1A), both groups had worsened performance during dual task conditions (cognitive with simultaneous motor element) compared to single (cognitive only) of 6.96 ± 1.71% for TD and 12.89 ± 1.5% DCD (TD: F_1,505_ = 16.59, *p* < 0.001***, *d* = *0.57* and DCD: F_1,505_ = 76.63, *p* < 0.001***, *d* = *0.95*). TD and DCD groups performed similarly (*p* > 0.05) on the cognitive only task, however with the addition of the motor element (dual task) the DCD group had 5.85 ± 1.61% worse performance (F_1,505_ = 13.2, *p* < 0.001***, *d* = 0.45).

#### Physical performance (PhysP)

Results regarding Physical Performance (PhysP), indicated no main effect for Group (F_1,71_ = 0.004, *p* = 0.95) between TD and DCD, but did indicate a significant main effect for Task (F_1,70.8_ = 5.40, *p* = 0.023*, *d* = *0.26*), between Single and Dual. Furthermore, no significant interaction effect between group and task was found within PhysP (F_1,70.8_ = 2.96, *p* = 0.090), as depicted in Fig. 1B.

### Neuroimaging results

**Figure 2:**
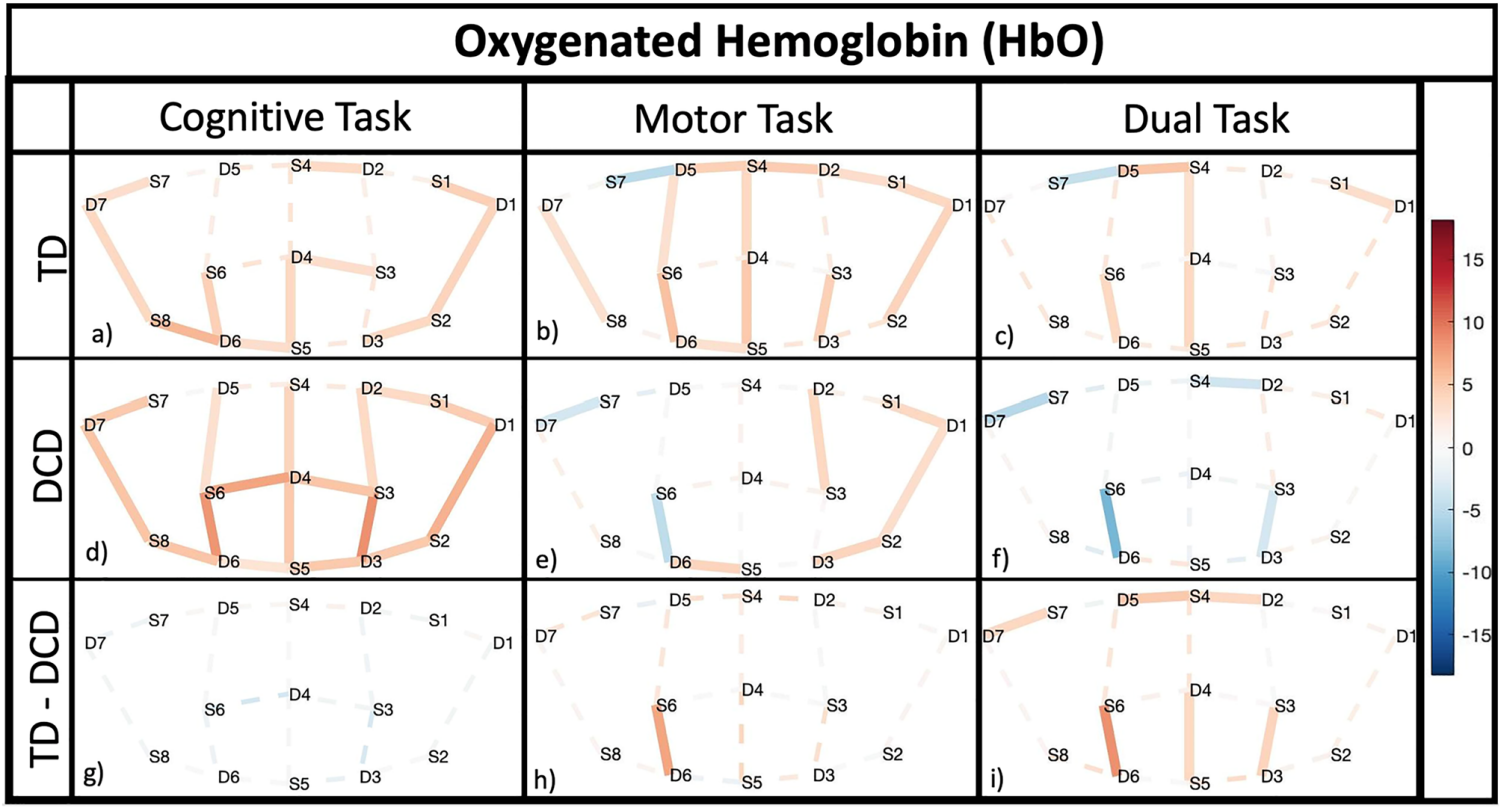
Neuroimaging and Neurobehavioral results for 85 subjects, displaying areas of interest across the PFC comparing differences between groups (TD of DCD) and task (Cognitive, Motor, or Dual Task). fNIRS results are displayed per group (rows 1&2) and task (columns 1–3), and between group (row 3). Red bars indicated increased HbO (activity), while blue bars represent decreased HbO according to international 10–10 system. Each group and task are assigned a specific legend (a through i) for reference within the results/discussion.

In localizing and evaluating motor-cognitive deficits of DCD during physical activity within the prefrontal cortex (PFC), we quantified the hemodynamic activation as it occurred during the tasks. Neuroimaging results are depicted in Fig. 2, displaying brain activity as measured via twenty optode measurement locations covering the PFC, per group and task.

The cognitive (non-motor) task elicited increased activity across many regions of the PFC for both TD and DCD children, with no significant regions of difference between the groups (*p*’s > 0.05).

The motor task elicited increased PFC activity for TD children, but significantly less so for DCD children within channel 16 (t(660) = 6.0695, *p* < 0.001, d = 0.9998) found in the right middle frontal gyri (mFG_R_).

The dual task led to significantly increased activity for TD children (Fig. 2c), while the DCD children approached the task with significantly reduced activity (Fig. 2f). The contrast between the groups highlighted six channels (mFG and sFG) of interest. Complementary information regarding HbR is depicted in Figure S1 within supplementary information.

### Combinatory/neurobehavioral results

Neural Efficiency (NE) relates the neurophysiological measures of brain activity to an individual’s performance according to the demands of the task and the capability of the individual^44^ in a combinatory measure for the evaluation of neurobehavior. The NE for both CogP and PhysP was evaluated for effects on Group, Task, and the interaction between Group and Task.

#### Neural efficiency of cognitive performance (NE of CogP): main effects

The main effect for Group was negligible for NE of CogP. However, Task condition indicated a significant main effect on 18/20 channels for NE of CogP indicating that dual tasking reduced NE as detailed in Table 1.

**Table 1:** Neural efficiency of cognitive performance (main effects).

Channel	Source–detector	Region	MNI coordinates			Distance (mm)	Specificity (%)	Mean difference	F-statistic	*p* (FDR corrected)	Effect size (d)
			X	Y	Z						
**Task: single > dual**											
1	F3–F5	mFG_L_	− 45	35	23	29	74.22	0.9096	33.562	< 0.001***	0.569
2	F3–F1	mFG_L_	− 30	38	39	29	87.01	0.3705	4.09	0.047*	0.227
3	AF7–F5	iFG_L_	− 47	42	4	34	87.56	0.9203	59.515	< 0.001***	0.750
4	AF7–Fp1	iFG_L_	− 34	56	− 4	31	53.57	0.7646	27.200	< 0.001***	0.556
5	AF3–F1	mFG_L_	− 24	50	30	44	80.24	0.7676	46.854	< 0.001***	0.704
6	AF3–Fp1	mFG_L_	− 26	60	5	30	90.79	0.6913	18.822	< 0.001***	0.393
7	AF3–AFz	mFG_L_	− 16	59	21	39	55.88	1.0458	54.451	< 0.001***	0.754
8	Fz–F1	sFG_L_	− 11	40	47	30	74.89	0.3706	4.465	0.038*	0.225
9	Fz–AFz	sFG_L_	0	48	37	40	48.54	0.67	47.326	< 0.001***	0.650
10	Fz–F2	sFG_R_	11	40	48	28	75.09	0.3892	5.572	0.021*	0.257
11	Fpz–Fp1	mFG_L_	− 14	64	− 3	31	50.16	0.7073	15.908	< 0.001***	0.395
12	Fpz–AFz	sFG_L_	− 1	61	11	41	47.28	1.3175	68.787	< 0.001***	0.867
13	Fpz–Fp2	mFG_R_	14	65	− 3	30	51.58	0.7740	42.650	< 0.001***	0.656
14	AF4–AFz	mFG_R_	15	59	22	37	52.67	0.9959	49.773	< 0.001***	0.727
15	AF4–F2	mFG_R_	23	51	31	43	75.53	0.6090	21.548	< 0.001***	0.476
18	F4–F6	mFG_R_	46	38	24	28	87.56	0.9374	21.350	< 0.001***	0.515
19	AF8–Fp2	iFG_R_	34	58	− 4	30	52.77	0.6259	13.432	< 0.001***	0.399
20	AF8–F6	iFG_R_	47	45	4	33	88.89	0.9287	59.580	< 0.001***	0.755
**Interaction between group and task**											
8	Fz–F1	sFG_L_	− 11	40	47	30	74.89	–	4.126	0.046*	–
9	Fz–AFz	sFG_L_	0	48	37	40	48.54	–	4.425	0.039*	–
12	Fpz–AFz	sFG_L_	− 1	61	11	41	47.28	–	5.462	0.022*	–
20	AF8–F6	iFG_R_	47	45	4	33	88.89	–	4.758	0.032*	–

Significant Channels (at Source–Detector locations) using MNI coordinates (X, Y, and Z), with the region designation of main effects for Neural Efficiency of Cognitive Performance for Group, Task, and Interaction. Specificity/Coverage of the region per channel is detailed, with the mean difference for the comparison (not applicable in interaction between Group and Task), along with statistical information (F-statistic, *p*-value, and effect size [not applicable for Interaction between Group and Task]). (*p* < 0.05*, *p* < 0.01**, *p* < 0.001***).

#### NE of CogP: interaction effects

**Figure 3:**
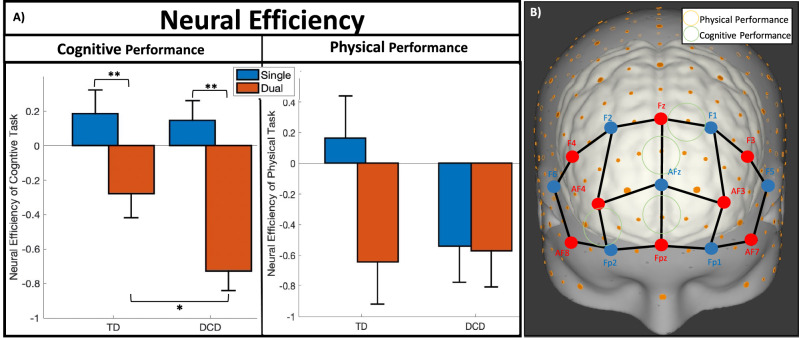
(**A**) Example patterns of Neurobehavioral (Neural Efficiency) results per group and task (*p < 0.05, **p < 0.01, ***p < 0.001) with error bars representing standard error of the mean. Cognitive Performance example pattern is from optode 9, while Physical Performance example pattern is from optode 13. (**B**) Significant regions of the Neural Efficiency patterns found in A, where green circles indicate significant patterns for the NE of Cognitive Performance, while the yellow circles indicate significant patterns for the NE of Physical Performance. There are no regions of significant interaction effect of group and task for NE of PhysP.

As detailed within Table 1, significant interaction effects were found in 4/20 channels for NE of CogP. Example patterns of the significant interaction between the factors of Group and Task are depicted in Fig. 3 for NE of CogP (i.e. channel 9 found in the sFG_L_) and further detailed in Table 2. Both groups had significantly decreased NE of CogP for the dual task condition compared to single (TD: 4 channels; DCD: the same four and two additional channels). Furthermore, during the cognitive only task (single condition), TD and DCD children had similar NE of CogP (*p* > 0.05), but with the additional simultaneous motor task (dual condition), the DCD group had significantly decreased NE of CogP compared to the TD group (4 channels). These patterns were evident across four channels within the PFC (mFG and sFG) (see Fig. 3 and Table 2).

**Table 2:** Neural efficiency (interaction effects of group and tasks).

Neural efficiency of cognitive performance											
Channel	Source–Detector	Region	MNI coordinates			Distance (mm)	Specificity (%)	Mean difference	F-statistic	*p* (FDR corrected)	Effect size (d)
			X	Y	Z						
**Typically developed: single > dual**											
9	Fz–AFz	sFG_L_	0	48	37	40	48.54	0.4651	9.584	0.006**	0.32
12	Fpz–AFz	sFG_L_	− 1	61	11	41	47.28	0.949	15.2126	< 0.001***	0.47
14	AF4–AFz	mFG_R_	15	59	22	37	52.67	0.7463	11.7451	0.002**	0.41
20	AF8–F6	iFG_R_	47	45	4	33	88.89	0.6663	12.8857	0.001**	0.39
**Developmental coordination disorder: single > dual**											
8	Fz–F1	sFG_L_	− 11	40	47	30	74.89	0.7269	10.6007	0.003**	0.36
9	Fz–AFz	sFG_L_	0	48	37	40	48.54	0.8749	49.8033	< 0.001***	0.64
12	Fpz–AFz	sFG_L_	− 1	61	11	41	47.28	1.6861	70.5415	< 0.001***	0.89
14	AF4–AFz	mFG_R_	15	59	22	37	52.67	1.2454	48.0464	< 0.001***	0.72
16	AF4–Fp2	mFG_R_	26	61	6	30	91.67	0.823	6.9152	0.019*	0.31
20	AF8–F6	iFG_R_	47	45	4	33	88.89	1.1912	60.491	< 0.001***	0.74
**Dual task: typically developed > developmental coordination disorder**											
8	Fz–F1	sFG_L_	− 11	40	47	30	74.89	0.6817	6.4882	0.024*	0.32
9	Fz–AFz	sFG_L_	0	48	37	40	48.54	0.4487	6.2352	0.028*	0.32
12	Fpz–AFz	sFG_L_	− 1	61	11	41	47.28	0.7273	8.5226	0.008**	0.37
16	AF4–Fp2	mFGR	26	61	6	30	91.67	0.8666	6.2113	0.028*	0.32

Significant Channels (at Source–Detector locations) using MNI coordinates (X, Y, and Z), with the region designation of Neural Efficiency of Cognitive Performance for the Interaction Effect of Group and Task between Typically Developed (TD) and Developmental Coordination Disorder (DCD) children and tasks (Single and Dual). Specificity/Coverage of the region is depicted, with the mean difference for the comparison, along with statistical information (F-statistic, *p*-value, and effect size). (*p* < 0.05*, *p* < 0.01**, *p* < 0.001***). No significant channels were implicated during Single Task, comparing TD and DCD children, nor for the interaction effects of NE of PhysP.

#### Neural efficiency of physical performance (NE of PhysP)

The main effect for group was negligible (*p*’s > 0.05) for the NE of PhysP in all channels. However, task condition indicated a significant main effect on 6/20 channels for NE of PhysP, indicating that dual tasking reduced NE as detailed in Table 3.

**Table 3:** Neural efficiency of physical performance (main effects).

Channel	Source–detector	Region	MNI coordinates			Distance (mm)	Specificity (%)	Mean difference	F-statistic	*p* (FDR corrected)	Effect size (d)
			X	Y	Z						
**Task: single > dual**											
4	AF7–Fp1	iFG_L_	− 34	56	− 4	31	53.57	0.386	5.578	0.021*	0.224
5	AF3–F1	mFG_L_	− 24	50	30	44	80.24	0.3268	4.779	0.032*	0.218
9	Fz–AFz	sFG_L_	0	48	37	40	48.54	0.3038	4.051	0.048*	0.207
17	17: F4–F2	mFG_R_	29	40	40	29	82.62	0.3616	4.308	0.042*	0.195
18	F4–F6	mFG_R_	46	38	24	28	87.56	0.3159	4.502	0.038*	0.228
19	AF8–Fp2	iFG_R_	34	58	− 4	30	52.77	0.3567	3.974	0.050*	0.165

Significant Channels (at Source–Detector locations) using MNI coordinates (X, Y, and Z), with the region designation of main effects for Neural Efficiency of Physical Performance for Group, Task, and Interaction. Specificity/Coverage of the region per channel is detailed, with the mean difference for the comparison (not applicable in interaction between Group and Task), along with statistical information (F-statistic, *p*-value, and effect size [not applicable for Interaction between Group and Task]). (*p* < 0.1^X^, *p* < 0.05*, *p* < 0.01**, *p* < 0.001***).

Additionally, no significant interaction effects (*p*’s > 0.05) were found in any of the twenty channels for NE of PhysP. An example patterns displaying data between the factors of group and condition are depicted in Fig. 3A for NE of PhysP (i.e. channel 13 found in mFG_R_).

## Discussion

The present study is the first ecologically relevant investigation of neurobehavioral differences of young people with developmental coordination disorder (DCD) using both a cognitive and a gross motor task. It employed a cross-sectional, within-subjects repeated measures design, with one of the largest neuroimaging cohorts, in which participants engaged in novel solitary cognitive, and motor tasks, and a dual task (where cognitive and motor were combined). The main findings are that: 1) behaviorally, while both DCD and TD children had reduced CogP in dual task conditions compared to single task, DCD children had significantly reduced dual task CogP compared to TD children, 2) neurally, as tasks became more complex (dual task)/had a motor element, differences in neural hemodynamics were elicited between the groups indicating that DCD children had a hypo-functioning PFC within the mFG and sFG, and lastly 3) neurobehaviorally, while both groups were neurally inefficient in dual task performance of CogP compared to single task, DCD children were significantly more neurally inefficient compared to TD children in the dual task. These results are in line with our hypotheses as well as what was expected in the literature.

### Behaviorally

As the results indicated, behaviorally, DCD and TD children showed significant differences of CogP specifically during dual tasking. While both groups generally had decreased performance in dual task compared to single, it was more significant for individuals with DCD. Both groups performed similarly, and well in the Stroop task, but when presented with a simultaneous physical challenge (the stepping task), the DCD group had significantly reduced performance on the Stroop task compared to the TD group, suggesting that this group were unable to maintain their physical task automaticity, a finding similar to that found in individuals after a stroke^51^. Therefore, in the case of dual task, children with DCD were unable to maintain their CogP while maintaining their PhysP, indicating that they may shed their cognitive task burden to maintain PhysP with their full attention, while TD children did not have to sacrifice as much of their CogP to maintain their PhysP. Furthermore, DCD children had neurotypical trends regarding task conditions (albeit reduced Cognitive Performance within dual task compared to TD children), but both TD and DCD children had similar (nonsignificant, *p* > 0.05) results for PhysP regardless of single or dual task condition. This alone suggests that when presented with a typical task that is purely cognitive (non-motor), having DCD does not impact performance. But when a motor component is added (dual task), the DCD group is much less capable than neurotypical children in CogP, emphasizing the impact of DCD as a motor-cognitive disorder.

### Neurally

These are some of the first neural activity findings involving a gross motor task for those with DCD. As predicted by the literature^1,6,52^, the DCD group showed increased neurological deficits/hypo-functioning as the tasks became more motor oriented and more complex. DCD children are neurally deficient with the introduction of motor tasking (particularly within the mFG and sFG), but otherwise cognitively equivalent to TD in non-motor tasks, highlighting the motor-cognitive deficiency found only during ecologically relevant settings and whole-body motor tasks.

Through neuroimaging, we were able to detect a clear difference in PFC activation between groups across tasks, where the number of optodes with significant differences between the groups grew as the task moved from single cognitive task to a single motor task, and finally to a dual task (0, 1, and 6 optodes respectively with increased HbO for the TD group). Optical neuroimaging results suggest increased inability to meet cognitive task demands for the DCD group as the tasks became more motor oriented, and more complex. This indicates that DCD children were impeded in activating certain regions of the PFC, and therefore resulted in reduced CogP during the dual task. Furthermore, while PhysP did not elicit group differences, neuroimaging may be more sensitive to determine group differences during single motor tasks.

### Neurobehaviorally

By combining the task performance and neuroimaging information we were able to show differences in neural efficiency generated for both the Cognitive and Physical Performance. As was predicted, both groups approached dual task with reduced neural efficiency compared to single task for CogP across much of the PFC (18/20 channels), with the DCD group being significantly more inefficient (particularly within the sFG_L_ and mFG_R_). During the dual task, individuals with DCD had decreased brain activity, alongside decreased cognitive task performance, and maintained physical task performance, leading to more neural inefficiency than the TD group. The results suggest increased task demand for the DCD group while being unable to generate an appropriate cortical response as the tasks became more motor oriented, and more difficult^1,6,52^. This is also similar in conditions including Attention-Deficit Hyperactivity Disorder^53^, Human Immunodeficiency Virus^54^, and Multiple Sclerosis^55^, where the acquired or inherited condition may not have led to significant differences in task performance or neuroimaging, but did lead to declined efficiency that was externally imperceptible clinically.

Interestingly, NE of PhysP did not reveal the same pattern but did elicit six regions of the PFC (as shown in Table 3) showing dual tasking was approached in a less efficient manner regardless of group. This may suggest that gross motor physical performance itself may not be as sensitive a measure as cognitive performance during dual tasking to elicit group or interaction differences and could be related to the subtlety of the motor deficiency found in DCD, which may only be appreciated during more demanding tasks.

These findings suggest that children with DCD are neuroergonomically impaired and experience increased difficulty when presented with a physical challenge, due to less efficient approaches in cognitive management for gross motor tasks. They highlight the importance of a combined neuroimaging and behavioral evaluation, and the possible outcomes for deficits. This may lead to the use of potential therapeutic strategies for improving motor performance in these children including brain stimulating devices (i.e. transcranial direct current stimulation, transcranial magnetic stimulation, etc.), physical therapies, or even pharmaceuticals^2,4,9,56–58^.

### Potential interventions

By aiming to localize and qualify the neural deficits within DCD, improving/optimizing neural activity through brain stimulating devices can potentially lead to improved cognitive and physical outcomes as has been demonstrated in memory deficits, depression, Parkinson’s disease, depression and many other previously difficult to localize neurological conditions^56–60^. Our findings also open the door for possible innovative drug and non drug approaches to increase activity in the prefrontal cortex and improve task acquisition and performance. Our data also suggests a mechanism for the enhanced difficulty of performing motor tasks in ecologically relevant settings for people with DCD that was previously unknown but similar in other conditions^53–55^. This methodological approach may detect mechanisms underlying changes in conditions creating possibilities for personalized and neuroadaptive interventions to transform outcomes for movement and balance disorders targeting regions of the PFC for neural engagement and recovery.

### Limitations and future work

There were several limitations to our study. While the group determined as DCD was very likely a DCD population meeting three of the four diagnostic criteria according to the DSM-5, they were not formally diagnosed. Furthermore, the MABC-2 does not differentiate between, or consider gender, and our DCD population had a larger proportion of girls than that of the TD group. Future studies may improve on our design and limitations by clinically diagnosing DCD and encouraging gender balancing. Additionally, while trying to maintain approximately similar fitness among TD and DCD children, the endurance measure was unbalanced, indicating increased Endurance ability for TD children. Additionally, while the physical task was chosen for its novelty, it may not have been a purely motor task as participants were requested to step on a visual instruction with their left or right leg [left on the left side and right on the right side] with a pattern that could be easily learned as there was some simple attentional cognitive demand to achieving task accuracy; further studies might employ more than one physical task with a range of cognitive overlap in their design. Furthermore, the use of NIRS, while advantageous in many regards in this study regarding comfort, motion artifact resistance, set-up time, etc., had limitations such that its signal sensitivity could only record cortical activity superficially, and was unable to monitor the entire surface of the brain due optode quantity restraints, and difficulty to monitor areas covered by thick hair^61^.

## Conclusion

This study is the first to reveal neural underpinnings of DCD during an active, gross motor task. Previously, due to constraints in neuroimaging, the literature only discussed DCD with respect to fine motor skills^1,8,9,11^. As in previous studies, our work further demonstrates that DCD is not an intellectual disability, but a motor learning and performance deficit (motor-cognitive disability) through both a neuroimaging and neuroergonomic lens^62^. Using new generation wearable and mobile optical neuroimaging that has been demonstrated to measure similar localized cortical hemodynamic activity as stationary and traditional neuroimaging^63–69^, we were able to localize functional deficits within the PFC of DCD children, which can allow for more targeted intervention. In addition, this approach provides a new perspective beyond clinical neuroscience, as the first study to use an applied combined human factors technique, to evaluate neural efficiency within DCD. The methods and approach demonstrated can be easily adapted to broader contexts within a host of disabilities that impact motor cognition in and out-of-laboratory settings. The approaches and results may be used in the future for triaging children for DCD, to accelerate diagnosis and assess therapeutic intervention as suggested in other developmental disorders^70^.

## Methods

### Participant recruitment

**Figure 4:**
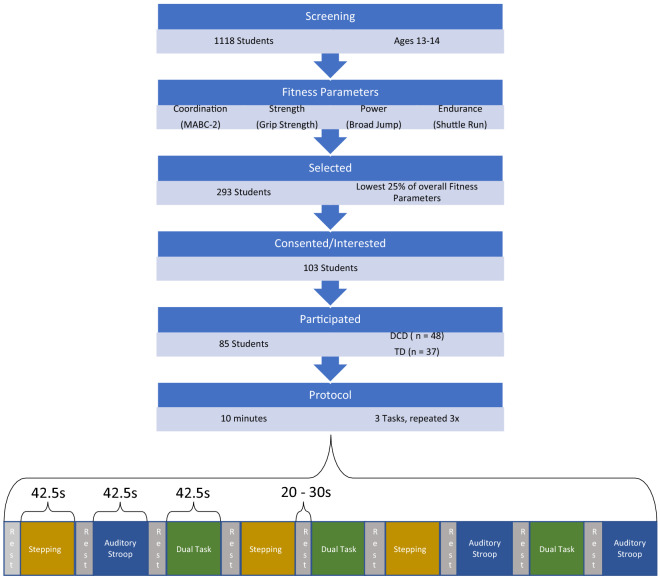
Procedure for participant selection and experimental protocol. Experimental flow, from screening for participants with Developmental Coordination Disorder through running the 10-min protocol. The 10-min indicates the three tasks (Motor Only Task [Stepping], Cognitive Only Task [Auditory Stroop], and the combinatory Dual Task [Auditory Stroop and Stepping simultaneously]) predicated by variable rests of 20–30 s repeated three times each, in a pseudo-random order.

Across 3 mainstream schools in Oxfordshire, a total of 1118 children (ages 13–14) screened for Developmental Coordination Disorder (DCD) using the Movement Assessment Battery for Children 2 (MABC-2) as detailed in Fig. 4. Fitness was controlled across both groups for using additional fitness parameters including strength^71^, power^72^, and endurance^73^, ensuring all groups were within the lowest quartile of these additional fitness parameters. Therefore, any group differences later found would be less drastic, but more confident, eliminating fitness as a confounding factor assessing specifically motor coordination. 293 students were eligible to join the study, of which 103 consented, and ultimately 85 students participated in the experiment.

Those that scored above the 15th percentile of the MABC-2 were identified as Neurotypical or Typically Developed (TD) and those below the 15th percentile were identified as likely DCD resulting in 37 TD and 48 DCD children^74–76^. Ultimately 85 children (ages 13.92 ± 0.33 yrs) participated in the study (33 male [54% TD and 27% DCD], and 10 left-handed [14% TD and 10% DCD]).

To formally diagnose a child with DCD, the Diagnostic and Statistical Manual (5th Edition) (DSM-V)^3^, specified clear criteria: 1) The acquisition and execution of coordinated motor skills is substantially below that expected given the individual’s chronological age and opportunity for skill learning and use; 2) The motor skills deficit significantly interferes with activities of daily living and impacts academic/school productivity, activities, leisure, and play; 3) onset of symptoms is in the early development period; and 4) the motor skills deficits are not better explained by intellectual disability, visual impairment or other neurological condition affecting movement. Within this study, criterion 1 was assessed using the MABC-2; criterion 2 was assessed by two methods including i) teachers to confirm each DCD child had observed motor shortcomings likely impacting academic/school, leisure or play activities, and ii) further evaluated for strength, power, and endurance (lowest quartile). Criterion 4 was evaluated through the consent process and confirmed with the parent/teacher to ensure no comorbidities or other explanations for motor skill deficit. Criteria 3 was the only criterion in the DCD diagnosis methodology not formally addressed, and therefore this study had a highly likely DCD cohort.

All participants were confirmed to meet the eligibility requirements, and did not have cognitive, neurological, musculoskeletal, behavioral, or non-correctable visual impairments based on self-report, parent, and teacher reports. The exclusion criteria specified impairments including but not limited to diagnosed conditions such as ADHD, Autism Spectrum Disorder, intellectual disorders/disabilities, Cerebral Palsy, Down Syndrome, etc. Control variables were gathered including height, weight, and puberty status (5-stage Tanner scale)^77,78^ as detailed in Table 4. Groups showed no significant differences between control variables. Prior to the study, all participants and respective guardians signed informed consent forms, and all methods were performed in accordance with the relevant guidelines and regulations approved by the University Research Ethics Committee (UREC Registration No: 161033) and the trial is registered under ClinicalTrials.gov (NCT03150784) on 12/05/2017. Informed consent was obtained from all subjects and or their legal guardian(s) for publication of the information, images and videos for an online open access publication.

**Table 4:** Group characteristics and comparisons.

Characteristic	DCD (n = 48)	TD (n = 37)	Statistics
Sex—no. (M/F)	13 M/35F	20 M/17F	
Handed—no. (R/L)	43R/5L	32R/5L	
Age (year)	13.95 ± 0.34 [13.86–14.05]	13.92 ± 0.32 [13.35–14.50]	t(83) = 1.078 *p* = 0.284
Weight (kg)	60.60 ± 13.80 [56.5.8–64.85]	58.45 ± 12.82 [54.44–62.96]	t(83) = 0.734 *p* = 0.465
Height (m)	1.61 ± 0.078 [1.59–1.64]	1.65 ± 0.098 [1.62–1.68]	t(83) = − 1.867 *p* = 0.065
BMI (kg/m^2^)	23.23 ± 4.80 [21.88–24.68]	21.41 ± 3.94 [20.18–22.72]	t(83) = 1.865 *p* = 0.066
Leg length (cm)	86.01 ± 5.024 [84.55–87.42]	90.40 ± 6.025 [88.6–92.38]	t(83) = − 3.67 *p* < 0.001***
Shoe size (UK)	6.04 ± 1.82 [5.53–6.58]	6.70 ± 2.30 [6–7.46]	t(83) = − 1.479 *p* = 0.143
Tanner stage^a^	3.22 ± 1.04 [2.77–3.63]	3.29 ± 1.01 [2.84–3.7]	t(42) = − 0.221 *p* = 0.826
Strength (kg)	23.36 ± 59.96 [21.77–24.96]	24.29 ± 6.72 [22.09–26.5]	t(84) = − 0.071 *p* = 0.482
Power (cm)	89.47 ± 59.96 [72.06–106.88]	91.31 ± 77.17 [65.58–117.04]	t(83) = − 0.124 *p* = 0.902
Endurance (shuttles)	27.22 ± 11.01 [23.95–30.49]	34.71 ± 9.9 [31.31–38.11]	t(79) = − 3.17 *p* = 0.002**
MABC (percentile)	6.13 ± 3.07 [5.23–7.02]	38.84 ± 16.44 [33.44–44.25]	t(84) = − 13.51 *p* < 0.001***
Aiming/catching	12.83 ± 18.02 [7.6–18.07]	50.61 ± 24.59 [42.52–58.69]	t(84) = − 8.22 *p* < 0.001***
Manual dexterity	10.76 ± 13.26 [6.91–14.61]	29.37 ± 23.70 [21.58–37.16]	t(84) = − 4.6 *p* < 0.001***
Balance	20.95 ± 17.96 [15.73–26.16]	53.79 ± 22.89 [46.27–61.31]	t(84) = − 7.46 *p* < 0.001***

Baseline Characteristics of DCD and TD children with comparison. Data shown details mean ± standard deviation [95% Confidence Interval], along with t-statistic (each characteristic showed equal variances among both groups [Levene’s tests were nonsignificant], degrees of freedom, and *p*-value (*p* < 0.01**, *p* < 0.001***).

^a^Only 44 participants (23 DCD and 21 TD children) indicated their Tanner Stage as many children opted not to disclose this information.

### Task protocol

The experiment consisted of a ten-minute session depicted in Fig. 4. Using a within subjects repeated measures design, where participants completed three tasks consisting of a cognitive only task (auditory stroop), a motor only task (rhythmic stepping), and a dual task (simultaneous cognitive and motor task). The tasks had a duration of 42.5 s each, with variable 20-30 s rest between tasks. These tasks were repeated three times each and are known as blocks.

The cognitive task utilized was an auditory Stroop test^79^, presented auditorily with stimulus at 0.33 Hz for approximately 14 prompts as shown in Audio S1 (supplementary file)— no visual cue was present. The task prompts consisted of a voice presenting the words “High” or “Low” in either a high (400 Hz) or low (200 Hz) pitch for approximately 0.8-1 s. Of the 14 prompts, half (seven) were congruent (high pitch along with the word “high” or low pitch along with the word “low”) and the other half were incongruent (high pitch along with the word “low” or low pitch along with the word “high”). Participants were instructed to auditorily respond with the categorization of the pitch, and to ignore the word auditorily stated, as shown in Video S1 (found in supplementary files).

The motor/physical task was a rhythmic stepping task, with instructions displayed visually on a laptop via a customized LabView Program in front of the participant at 0.5 Hz. The stepping instructions consisted of the words “left” or “right” displayed on the left or right side of the screen respectively for a duration of 1.5 s for a total of twenty stimuli (equally left and right). These stimuli were displayed after a “get ready” cue of a duration of 2.5 s. When instructed “left”, participants had to step onto a standardized stepping block with their left foot initially followed by their right, then back down with their left foot followed by their right and vice versa for the “right” prompt.

The dual task involved the simultaneous auditory Stroop task and the rhythmic stepping task. Children were instructed to perform both cognitive and motor tasks simultaneously.

### Behavioral performance metrics

Cognitive Performance (CogP) was calculated as a correct percentage of the number of auditory Stroop trials per block and per participant. Auditory Stroop responses were manually recorded by research assistants, and later calculated as CogP.

Physical Performance (PhysP) was calculated using information generated from an Inertial Measurement Unit (IMU; LPMS-B2, Life Performance Research, Japan) fitted to lower back of each participant (measuring physical activity at 100 Hz). The IMU comprised of tri-axial accelerometers, gyroscopes and magnetometers, synced to the visual stimulus of the motor/physical task, recording physical activity per participant and repetition block. The feature extraction of the IMU data was calculated as adherence to the stimulus frequency (0.5 Hz), where perfect in rhythm synchronization was 100%, while any difference to the stimulus would decrease PhysP. Therefore, PhysP was calculated as the percentage of the ratio of Block Frequency to Stimulus Frequency.

Both behavioral measures of CogP and PhysP were calculated per repetition block, per participant and calculated within the respective tasks (cognitive and physical tasks) and within the dual task (combination of both tasks). Higher value indicates higher accuracy. Ideal performance (performance of 100%) quantified performance without any error (for CogP) or without any difference from the stimulus frequency (PhysP).

### Neural activity acquisition

Each participant was fitted with a portable and battery operated fNIRS sensor (NIRSport, NIRx Medical Technologies LLC, Glen Head, NY, USA) positioned over the forehead. fNIRS channel placement was standardized according to the established international 10–20 system for the eight light source and seven detector placements as depicted in Fig. 3B. Cortical regions with landmarks for the experimental configuration were generated using fNIRS Optodes Locator Decider (FOLD) toolbox^80,81^ with the Laboratory of Neuroimaging (LONI) Probabilistic Brain Atlas (LPBA40)^82^. Table 5 shows each channel according to source-detector pair Electroencephalography (EEG) labeling with ‘x–y-z’ configuration coordinates and brain area/landmark specificity for improved comparability and reproducibility. The inter-channel distance of approximately 3 cm formed 20 channels (measurement areas) sampled at 7.8125 Hz.

**Table 5:** fNIRS positions and brain locations.

Channel_(S:D)_	Source	Detector	Brain area	Specificity (%)	MNI coordinates			D (mm)
					X (mm)	Y (mm)	Z (mm)	
1_(S1:D1)_	F3	F5	mFG_L_	74.22	− 45	35	23	29
2_(S1:D2)_	F3	F1	mFG_L_	87.01	− 30	38	39	29
3_(S2:D1)_	AF7	F5	iFG_L_	87.56	− 47	42	4	34
4_(S2:D3)_	AF7	Fp1	iFG_L_	53.57	− 34	56	− 4	31
5_(S3:D2)_	AF3	F1	mFG_L_	80.24	− 24	50	30	44
6_(S3:D3)_	AF3	Fp1	mFG_L_	90.79	− 26	60	5	30
7_(S3:D4)_	AF3	AFz	mFG_L_	55.88	− 16	59	21	39
8_(S4:D2)_	Fz	F1	sFG_L_	74.89	− 11	40	47	30
9_(S4:D4)_	Fz	AFz	sFG_L_	48.54	0	48	37	40
10_(S4:D5)_	Fz	F2	sFG_R_	75.09	11	40	48	28
11_(S5:D3)_	Fpz	Fp1	mFG_L_	50.16	− 14	64	− 3	31
12_(S5:D4)_	Fpz	AFz	sFG_L_	47.28	− 1	61	11	41
13_(S5:D6)_	Fpz	Fp2	mFG_R_	51.58	14	65	− 3	30
14_(S6:D4)_	AF4	AFz	mFG_R_	52.67	15	59	22	37
15_(S6:D5)_	AF4	F2	mFG_R_	75.53	23	51	31	43
16_(S6:D6)_	AF4	Fp2	mFG_R_	91.67	26	61	6	30
17_(S7:D5)_	F4	F2	mFG_R_	82.62	29	40	40	29
18_(S7:D7)_	F4	F6	mFG_R_	87.56	46	38	24	28
19_(S8:D6)_	AF8	Fp2	iFG_R_	52.77	34	58	− 4	30
20_(S8:D7)_	AF8	F6	iFG_R_	88.89	47	45	4	33

Channels and Source–Detector (S:D) locations using Montreal Neurological Institute (MNI) coordinates (X, Y, and Z), with the brain area designation according to International 10–20 system of source–detector location designation. Brain Areas include inferior, middle, and superior (i, m, s) regions of Frontal Gyrus (FG) in either Left or Right hemispheres (L or R). Last column represents distance between source and detector.

fNIRS data was recorded via NIRStar (v14.0) and processed via NIRS AnalyzIR toolbox^83,84^. For each participant, attenuation changes in raw light intensity fNIRS data (two wavelengths of 850 nm and 760 nm) were transformed to concentration changes of oxygenated (HbO) and deoxygenated (HbR) hemoglobin respectively using the modified Beer-Lambert approach^85^. The data were pre-whitened to resolve high frequency noise, cardiovascular effects, and signal drift using an autoregressive model^83^. A baseline correction algorithm designed to remove motion artifacts/DC shifts was applied^84^, followed by a wavelet filter to remove motion artifacts with a threshold of 5 standard deviations, and a basis function of sym8^86^.

Beta values were calculated from HbO/HbR amplitudes for each block with local baseline (paired t-test: rest vs. circuit) per source-detector pair or channel for each task condition through subject-level autoregressive iteratively reweighted least squares General Linear Modeling. The parameter estimates were derived using a canonical Hemodynamic Response Function (HRF), as previous evidence suggests that tasks of duration longer than ten seconds, such as within this experiment, have better performance for testing hypothesis of difference response amplitudes^87^. The parameters of the canonical (double gamma function) HRF employed included: 1 s as the dispersion time constants for the peak and undershoot period, 4 s and 16 s as the peak and undershoot time respectively, 1:6 as the ratio of main peak height to the undershoot, and 32 s as the duration.

### Neural efficiency extraction

Neural Efficiency (NE) relates the neurophysiological measures of brain activity to an individual’s performance according to the demands of the task and the capability of the individual^44^. NE calculations incorporated the Neural Metrics (HbO) with the Behavioral Performance metrics (Cognitive Performance and Physical Performance) within the formula below resulting in NE of CogP and NE of PhysP respectively.

$$NE = \frac{{z\left( {Behavioral \;Performance} \right) - z\left( {Neural \;Metric} \right)}}{\sqrt 2 }$$

NE was calculated using the z-score of Behavioral Performance and the Neural Metric per individual trial against the entirety of the sample population and average of all trials regardless of task condition (i.e. z(Cognitive Performance) − z(HbO) and z(Physical Performance) − z(HbO))^44^.

### Statistical approach and analysis

Statistical analysis of behavioral performance metrics (CogP and PhysP) during the experimental procedure employed the use of Linear Mixed Modeling (LMM) implemented in NCSS (NCSS, LLC. Kaysville, Utah, USA). The dependent measures were assessed, and parameter estimates derived. Bonferroni *p*-value adjustments were calculated to indicate significance for interaction effects. Cohen’s d values were also calculated to indicate the observed effect size. The subject factor was treated as a random effect while the fixed effects were group (TD vs. DCD) and task condition (single vs. dual).

Within the neuroimaging results, group analysis employed mixed effects with repeated measures across the entire sample allowing for a population inference of the neural measures (HbO and HbR) per channel. The subject factor was treated as a random effect while the fixed effects were group (TD vs. DCD), and task (Auditory Stroop vs. Stepping vs. Dual). Type I Errors were controlled using false detection rate (FDR) Benjamini–Hochberg adjustments^88,89^.

Statistical analysis of NE metrics (NE of CogP and NE of PhysP) per channel following the approach of that of performance metrics. The subject factor was treated as a random effect while the fixed effects were group (TD vs. DCD) and task condition (single vs. dual).

## Supplementary Information

Supplementary Audio 1.

Supplementary Information 1.

Supplementary Video 1.

## Data Availability

All data are available in the main text, or the supplemental material.
