# Supplementary material for: Neuroergonomic assessment of developmental coordination disorder
Source: Sci Rep. 2022 Jun 17;12:10239. doi: 10.1038/s41598-022-13966-9 (PMC9206023; doi:10.1038/s41598-022-13966-9)
Supplement: Supplementary file 2 — Supplementary Information 1. [file 41598_2022_13966_MOESM2_ESM.pdf]

Supplemental Information

for

Neuroergonomic Assessment of Developmental Coordination Disorder

Shawn Joshi[1-4*] Benjamin D. Weedon[3,4], Patrick Esser[3,4], Yan-Ci Liu[3-5], Daniella N. Springett[3,4,6], Andy Meaney[3], Mario Inacio[3,7], Anne Delextrat[3], Steve Kemp[3], Tomas Ward[8], Hooshang Izadi[9], Helen Dawes[3,4], Hasan Ayaz[1,10-13]

*Corresponding Author: sj633@drexel.edu

[1]School of Biomedical Engineering, Science and Health Systems, Drexel University, Philadelphia PA, United States.

[2]College of Medicine, Drexel University, Philadelphia PA, United States.

[3]Centre for Movement, Occupation and Rehabilitation Services, Oxford Brookes University, Oxford, United Kingdom

[4]Nuffield Department of Clinical Neurology, University of Oxford, Oxford, United Kingdom

[5]School and Graduate Institute of Physical Therapy, College of Medicine, National Taiwan University, Taipei, Taiwan

[6]Department for Health, University of Bath, Bath, United Kingdom

[7]Research Center in Sports Sciences, Health Sciences and Human Development, University Institute of Maia, Porto, Portugal

[8]Insight SFI Research Centre for Data Analytics, Dublin City University, Dublin, Ireland

[9]School of Engineering, Computing and Mathematics, School of Technology, Design and Environment, Oxford Brookes University, Oxford, UK

[10]Department of Psychology, College of Arts and Sciences, Drexel University, Philadelphia, PA, United States

[11]Drexel Solution Institute, Drexel University, Philadelphia, PA, United States

[12]Department of Family and Community Health, University of Pennsylvania, Philadelphia, PA, United States

[13]Center for Injury Research and Prevention, Children's Hospital of Philadelphia, PA, United States

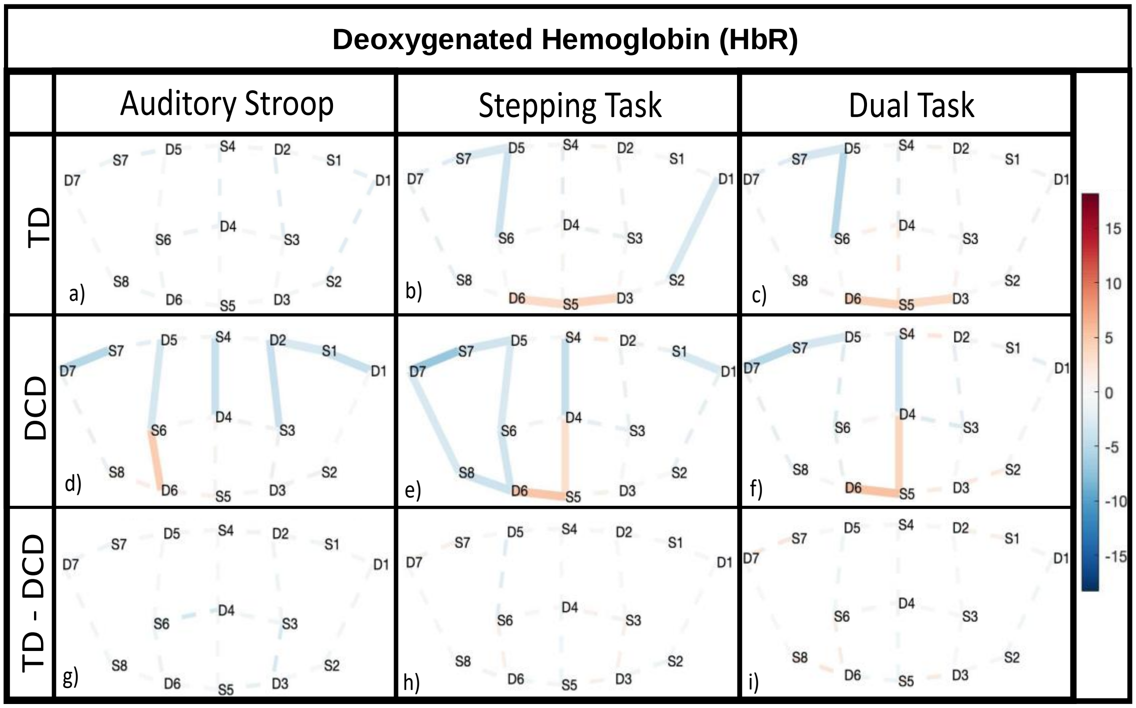

Fig. S1. Neuroimaging and Neurobehavioral results for 85 subjects, displaying areas of interest across the PFC comparing differences between groups (TD of DCD) and task (Cognitive, Motor, or Dual Task). A) fNIRS results per group and task, and between group (red box). Red bars indicated increased HbR (decreased activity), while blue bars represent decreased HbR according to international 10-10 system.

Movie S1. Experimental Setup during Active Task

Experimental Setup with example participant during partial Cognitive, Motor, and Dual Tasks. The auditory responses were to the auditory stroop task (Audio S1), while the stepping patterns were responsive to the onscreen instructions. The participant was wearing the functional near infrared spectroscopy device during the experiment.

Audio S1. Auditory Stroop File

Auditory Stroop test stated by individual. The auditory file revealed 14 trials per participant (7 Lows and 7 Highs) with equal congruent and incongruent trials.
